# Development and Validation of the Bone Tumor Surgery Complexity Score

**DOI:** 10.3390/reports7020035

**Published:** 2024-05-10

**Authors:** Annika Frei, Georg Schelling, Philip Heesen, Pietro Giovanoli, Bruno Fuchs

**Affiliations:** 1 Tumorzentrum, Kantonsspital Winterthur, 8400 Winterthur, Switzerland; 2 LUKS University Hospital, Orthopedic Oncology, Klinik für Orthopädie und Unfallchirurgie, Sarcoma Center, Faculty of Health Sciences and Medicine, University of Lucerne, 6000 Lucerne, Switzerland; georg.schelling@luks.ch; 3 Medizinische Fakultät, Universität Zürich, 8032 Zurich, Switzerland; 4 Klinik für Plastische Chirurgie und Handchirurgie, UniversitätsSpital Zürich, 8091 Zurich, Switzerland; 5 Sarcoma Center, Klinik für Orthopädie und Traumatologie, Kantonsspital Winterthur, 8400 Winterthur, Switzerland

**Keywords:** bone tumors, complexity score, bone sarcoma, benchmarking, value-based healthcare, quality indicator, surgical planning, validation

## Abstract

Bone tumors often manifest with non-specific symptoms such as pain and swelling, often posing diagnostic challenges. Optimal treatment requires centralized care in specialized centers, emphasizing the need for complete tumor removal and interdisciplinary collaboration. We developed the Bone Tumor Surgery Complexity Score (BT-SCS) based on a retrospective study of 501 patients. The BT-SCS, structured around patient demographics, tumor biology, and surgical parameters, categorizes surgical cases into four groups to comprehensively assess complexity. Application of the BT-SCS resulted in scores ranging from 3 to 33, with an average score of 14 ± 7.2. Patients with malignant tumors had higher scores (19.6 ± 5.2) compared to those with benign (10.0 ± 3.8) or intermediate malignant tumors (14.6 ± 7.1). Patients with pelvic tumors registered the highest scores (16.0), followed by extremities/trunk (14.3) and spinal tumors (13.6). The BT-SCS was validated against the Case Mix Index (CMI), using an independent cohort of bone and soft tissue cases. This validation process, utilizing Loess smoothing, illustrated the BT-SCS’s granular differentiation of surgical complexity, particularly in the lower-to-mid-range of case severities. The BT-SCS represents a significant shift from volume-based to complexity-based assessments in surgical care, aligning with evolving healthcare paradigms. It serves as a tool for strategic patient allocation to treatment centers, aiming to improve outcomes and benchmarking in sarcoma care. The score’s development and application in clinical practice align with the focus on patient-centered and value-based healthcare. Future enhancements, including machine learning integration and outcome data, will refine its categorization process, enhancing clinical utility.

## 1. Introduction

Bone tumors represent a challenging aspect of oncology, characterized by their rarity and the subtlety of their clinical presentation [1]. Patients often exhibit non-specific symptoms such as pain, swelling, or fractures, if any symptoms at all, making early detection and diagnosis challenging [2]. The differentiation between benign and malignant bone tumors is further complicated by the overlap of symptoms with other conditions, necessitating advanced radiological and histological examination methods for accurate diagnosis.

Surgical intervention is pivotal in the management of bone tumors, aiming for complete resection with tumor-free margins to ensure optimal patient outcomes. Given the primary occurrence of bone sarcomas in the extremities, alongside their potential to originate from any bone or soft tissue, a multidisciplinary approach is crucial [3]. It is widely recognized that patients benefit significantly from treatment at specialized sarcoma networks, where comprehensive care and expertise are available to address the unique challenges posed by these tumors [4].

Historically, the relationship between surgical volume and patient outcomes has been a topic of considerable research, with higher-volume centers often achieving better results [5]. This correlation suggests a benefit to centralizing care; however, it also underscores the complexity of attributing outcomes solely to volume, as it may not adequately reflect the expertise of individual surgeons or account for the multifaceted nature of surgical procedures [6,7,8]. Recent studies have begun to explore beyond volume, suggesting that a detailed understanding of surgical complexity and patient-specific factors is essential for optimizing care [9,10,11].

In this context, we previously developed the “Soft Tissue Sarcoma Surgery Complexity Score (STS-SCS)” to quantify surgical interventions’ complexity in soft tissue tumors [12]. Building on this work, the current study introduces the “Bone Tumor Surgery Complexity Score (BT-SCS)”, aiming to extend the same rigorous evaluation to surgeries involving bone tumors. This novel scoring system is designed to provide a standardized method for assessing surgical complexity, incorporating factors such as tumor location, size, and the necessary surgical skills for successful intervention.

The BT-SCS is rooted in the principle that a comprehensive evaluation of both procedural complexity and the patient’s overall health status is crucial for determining the most appropriate care setting. This approach aligns with the shift towards value-based healthcare, which prioritizes outcomes and quality over volume [13,14]. By accurately assessing and benchmarking surgical complexity, we can better allocate resources, minimize patient wait times, and enhance the efficiency and effectiveness of care [15,16,17].

Furthermore, the BT-SCS facilitates a more nuanced understanding of the challenges inherent in bone tumor surgery. It underscores the importance of interdisciplinary collaboration, particularly in complex cases requiring advanced surgical techniques and postoperative care. Through this standardized complexity assessment, we aim to support surgeons in planning and executing procedures, ultimately improving patient outcomes and contributing to the broader goals of healthcare quality and cost efficiency.

## 2. Materials and Methods

### 2.1. Study Population

A fellowship-trained surgeon performed a total of 501 surgeries on bone tumors over a 15-year period. These surgical procedures were recorded in the “Sarcoma Surgeon’s Registry”, managed by Adjumed Services AG in Zurich, Switzerland (website: www.adjumed.ch, accessed on 30 May 2023). To conduct basic statistical analyses and extract data, the AdjumedAnalyze tool, provided by Adjumed Services AG, was used. Subsequently, individual scores were calculated using Microsoft Excel, a product of Microsoft Corporation that is based in Redmond, WA, USA. All patients included in this study were discussed at an interdisciplinary board, provided by the SwissSarcomaNetwork (website: https://www.swiss-sarcoma.net/, accessed on 13 September 2023).

### 2.2. Development of the Bone Tumor Surgery Complexity Score (BT-SCS)

**Figure 1:**
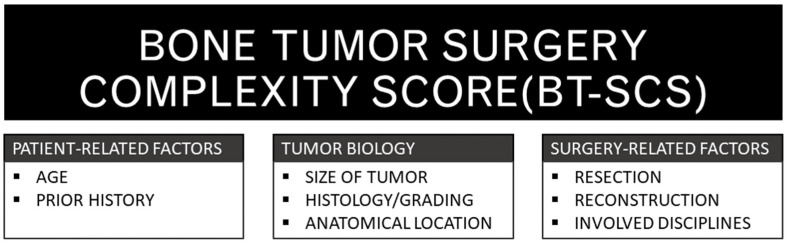
A schematic representation of the three foundational domains and their respective factors underpinning the BT-SCS.

Drawing from the literature and insights from seasoned sarcoma surgeons, we formulated the Bone Tumor Surgery Complexity Score (BT-SCS). This score was developed in a manner analogous to that of the Soft Tissue Sarcoma Surgery Complexity Score, which we previously delineated [12]. The BT-SCS is based on insights from the literature and experienced surgeons. It is fundamentally structured around three core domains—patient characteristics, tumor biology, and surgical parameters (Figure 1). The considerations that led to the content of the score are outlined below.

After establishing the foundational domains of the BT-SCS, we further refined the concept of surgical case complexity specifically for this study. This refinement involved operationalizing complexity in a measurable and replicable manner, ensuring that it encompasses the full scope of surgical challenges, from patient preparation and surgical planning to the execution of the procedure and postoperative management. This comprehensive approach allowed us to systematically evaluate and score the complexity of each surgical case individually, contributing to the robustness and applicability of the BT-SCS in clinical practice.

#### 2.2.1. Patient Characteristics

Factors pertinent to the patient, such as age and medical history, especially prior radiotherapy or chemotherapy, were incorporated, mirroring the approach in the STS-SCS. Notably, older osteosarcoma patients exhibit a poorer prognosis than their younger counterparts [18,19]. Studies indicate that osteosarcoma in individuals over 18 years is linked with a notably higher recurrence rate and worse outcomes [20]. However, surgical interventions in pediatric patients present unique challenges, due to their rarity and the specific requirements necessitated by the patient’s age, impacting both surgeons and anesthesiologists.

Historically, osteosarcoma patients faced grim prognoses, with many succumbing in a short span of time. However, advancements in surgical techniques and the advent of chemotherapy have revolutionized outcomes. A multidisciplinary approach has bolstered the rate of conservative procedures to 85% and enhanced the prognosis, with up to 70% 5-year disease-free survival (DFS) for nonmetastatic patients [21]. Neoadjuvant therapies, while beneficial, come with associated risks. For instance, neoadjuvant radiotherapy might impede wound healing, and chemotherapy could delay surgical intervention. Furthermore, improperly positioned biopsy channels outside the intended surgical zone can complicate bone tumor surgeries. Hence, a collaborative approach between radiologists and surgeons is paramount for optimal biopsy planning.

#### 2.2.2. Tumor Biology

Tumor size plays a pivotal role in surgical complexity, with larger tumors posing greater challenges [22]. Benign bone tumors, which seldom recur locally, are typically managed with complete excision or curettage [1]. Intermediate malignant bone tumors, characterized by aggressive local growth, necessitate wide excision. In contrast to benign tumors, they occasionally metastasize, predominantly to the lungs. Malignant tumors, while infrequent, have the propensity for distant metastasis in addition to local growth and recurrence. Bone sarcomas, contingent on histological type and grade, present a metastasis risk ranging from 20 to 100% [1]. Their local invasive growth makes malignant bone tumors particularly challenging to excise, in comparison to benign tumors. Certain bone tumors, especially those located in the spine or pelvis, are inherently challenging due to their deep anatomical positioning.

#### 2.2.3. Surgical Parameters

Surgical strategies vary based on tumor histology, location, and size. Meticulous planning is essential, especially when considering pelvic anatomy, with the objective of optimizing functional outcomes, while minimizing morbidity [23]. Such surgeries demand profound expertise from the entire medical team, particularly radiologists and surgeons. An interdisciplinary team, comprising orthopedic oncologists, medical oncologists, and radiation oncologists, is recommended for bone tumor management [24]. The intricate nature of bone tumor surgeries often necessitates collaboration across multiple surgical disciplines, including orthopedics, plastic surgery, and neurosurgery [25]. Post-excision, patients frequently undergo complex reconstructions. We have encapsulated this diverse range of surgical interventions within the BT-SCS, using a point-based system.

#### 2.2.4. Methodology and Rationalization of BT-SCS Scoring Criteria

In the development of the BT-SCS, each surgical parameter was evaluated and quantified based on a multifaceted approach, considering the effort, level of technical difficulty, time consumption, frequency, and skill requirement. This evaluation was substantially informed by a structured modified Delphi process, which involved extensive and iterative discussions over four rounds, among a diverse panel of expert sarcoma surgeons. These experts span the spectrum of orthopedic oncology, as well as reconstructive surgery, providing a broad and inclusive range of perspectives. The Delphi process was meticulously designed to foster open, critical dialogue and consensus-building, ensuring that the scoring criteria developed are deeply rooted in the practical and varied experiences of expert sarcoma surgeons.

It is important to note that while the BT-SCS offers a structured framework for assessing surgical case complexity, it inherently contains subjective elements and should be seen as a starting point for ongoing refinement. Throughout this process, the expert panel was tasked with identifying and defining the most impactful parameters that influence surgical complexity, leading to the prioritization of factors such as anatomical location and associated surgical risks. The score is designed to assess surgical case complexity in its purest form, independent of patient outcomes. As such, it aims to provide a baseline for surgical planning and resource allocation, with the understanding that future iterations will continue to evolve, based on clinical feedback and emerging surgical insights.

Additionally, while developing the BT-SCS, we recognized the inherent heterogeneity of bone tumors. Rather than focusing extensively on the histological diversity of these tumors, our scoring system is designed to emphasize the surgical challenges presented by their anatomical locations. This approach ensures that the BT-SCS remains broadly applicable and relevant across all types of bone tumors. It prioritizes the practical aspects of surgical intervention—such as accessibility, proximity of critical structures, and potential for surgical complications—which are crucial for assessing and planning complex surgical procedures. By concentrating on these surgical variables, the BT-SCS provides a standardized tool that assists in anticipating the complexities involved, irrespective of the tumor’s histological type.

#### 2.2.5. Conclusion

The BT-SCS amalgamates the multifaceted factors associated with bone tumor surgical resections into a comprehensive score (Table 1). Each factor is weighted based on its significance. We subsequently applied this score to a cohort of 501 patients, with data sourced from Adjumed.

### 2.3. Categorization of Complexity Scores

The categorization of surgical cases into four groups based on the BT-SCS was guided by a desire to create a practical and functional classification system for clinical use. Each category represents a distinct level of complexity, with approximately 25% of patients allocated to each group. This division was determined based on the score distribution in our study population and aimed to facilitate an easier interpretation and application of the BT-SCS in clinical settings. It is important to note that this categorization is a preliminary step, serving as a basis for potential clinical integration and future refinement. The division into quartiles is not intended to correlate directly with patient outcomes, but to provide a structured approach to understand and manage varying degrees of surgical case complexity in bone tumor treatment.

### 2.4. Statistics

Descriptive statistics were employed to analyze the data. Continuous variables, such as age and the Bone Tumor Surgery Complexity Score (BT-SCS), were summarized using measures of central tendency (mean) and dispersion (standard deviation). Categorical variables, like gender and tumor type, were presented as frequencies and percentages. The distribution of the BT-SCS across different tumor types and locations was visualized using appropriate graphical representations. Comparisons of BT-SCS scores across different tumor types were made using the mean and standard deviation values. All statistical analyses were conducted using Microsoft Excel (Version 16.78.3).

### 2.5. Validation of the Surgical Complexity Score against the Case Mix Index (CMI)

In our endeavor to establish a robust Surgical Complexity Score for soft tissue [12], and now extended to bone tumors, we recognize the necessity of validation against a well-established standard. The Case Mix Index (CMI), widely and internationally acknowledged for its significance in reflecting the complexity and resource requirements of hospital patient populations, offers an ideal benchmark. The CMI’s widespread utilization, especially in billing and reimbursement processes (based on case complexity) across various healthcare systems globally, underscores its relevance and robustness as a comparative standard. By aligning our newly developed complexity score with the CMI, we aim to affirm its validity and applicability in the clinical setting, thereby enhancing its potential for broader acceptance and implementation.

For the validation of our newly established Bone and Soft Tissue Tumor Surgery Complexity Scores (BS-/STT-SCS), we selected an (temporally) independent cohort of patients from our dedicated sarcoma treatment, Sarconnector^®^ warehouse (Table 2). This cohort, distinguished from the initial development set, encompasses a diverse array of bone and soft tissue sarcoma cases, meticulously documented and analyzed within our RWTD/E data warehouse [13]. By conducting a comparative analysis against the established CMI values within this independent patient set, we seek to rigorously assess the congruency and predictive accuracy of our complexity scores, using Locally Estimated Scatterplot Smoothing (LOESS). This step is crucial for establishing the reliability and clinical utility of the BT/STS-SCS, setting the stage for its integration into surgical planning and healthcare resource optimization for sarcoma care.

## 3. Results

### 3.1. Characteristics of Bone Tumor Patients

**Figure 2:**
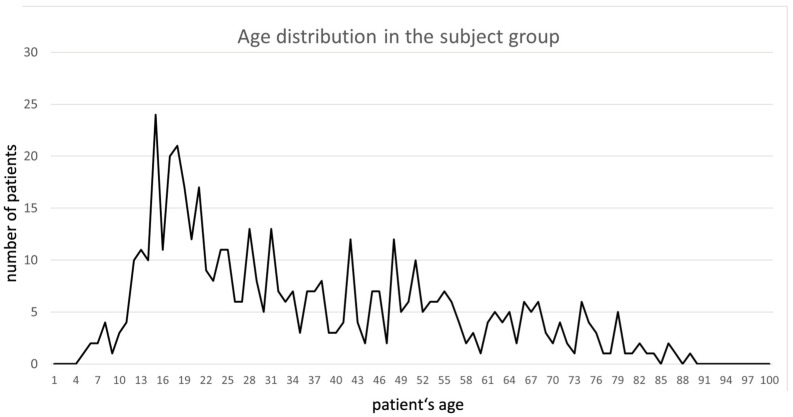
Age distribution in the subject group.

In this study, we investigated the surgeries of 501 patients with both benign and malignant diagnoses. In this patient population, the average age was 36.0 ± 20.1 years; the youngest patient was 5 years old and the oldest patient was 89. The expected first frequency peak of bone tumor diagnoses in adolescence and young adulthood is evident. The second peak found in the literature, which is supposed to occur in older adulthood, is not explicitly reflected in our data (Figure 2). Of the patients studied, 285 were male, representing a proportion of 57%, and 216 were female (43%). The male-to-female ratio was 1.32. Most patients operated on during this study had malignant tumors (180 patients, 36%), 148 had benign tumors (30%), and 107 had tumors with intermediate malignancy (21%). In total, 43 patients (9%) had metastases from other primary tumors. A minority suffered from tumor simulators (tumors which may imply a sarcoma on imaging, but are later revealed to be a benign lesion) and hematologic tumors of bone (incl. myeloma and lymphoma).

### 3.2. Application of the BS-SCS

**Figure 3:**
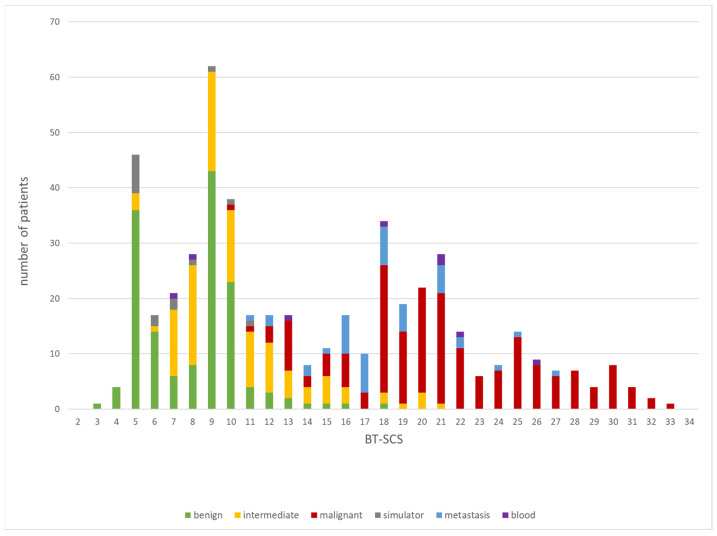
Distribution of the totals of the Bone Tumor Surgery Complexity Score (BT-SCS) in the sampling group.

**Figure 4:**
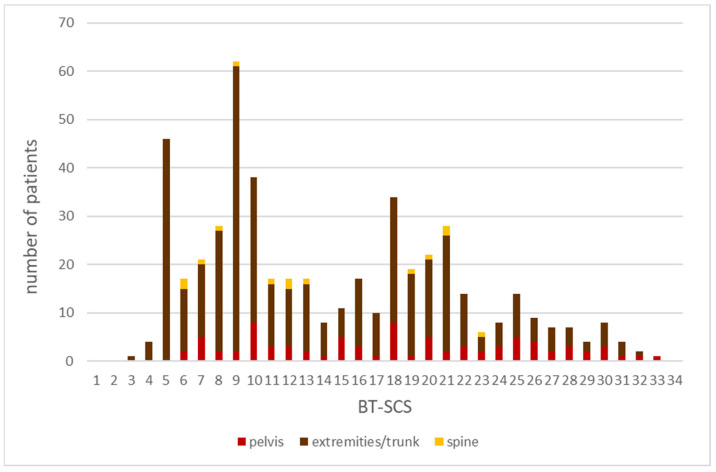
Distribution of the totals of the Bone Tumor Surgery Complexity Score (BT-SCS) in the sampling group, with respect to tumor localization.

The BT-SCS was applied to our sampling group of 501 patients and the individual scores were calculated for each patient using Microsoft Excel. The minimum score was 3 and the maximum score was 33 (out of 70), with an average score of 14 ± 7.2. The scores of patients with malignant diagnoses (19.6 ± 5.2) were notably higher than those of patients with benign (10.0± 3.8) or intermediate malignant tumors (14.6 ± 7.1) (Figure 3). Patients with tumors of the pelvis had the highest scores (16.0), followed by patients with tumors of the extremities and trunk (14.3) and patients with tumors of the spine (13.6) (Figure 4). The following examples illustrate these scores. The patient with a total score of 3 was a 42-year-old patient with no relevant prior history. He presented with a painful limitation of motion of the right knee. After an uneventful surgery, using curettage, by a single surgeon, single fragments of a benign finding, namely, synovial chondromatosis, could be detected histologically. The highest score of 33 was received by a 29-year-old patient who presented with an approx. 6 cm large, highly malignant, undifferentiated, high-grade pleomorphic sarcoma of the pelvic bone. After 3D planning, a type I internal hemipelvectomy was performed through the sacral ala, followed by complex reconstruction with instrumentation with rods and screws and a vascularized fibular autograft (based on fibular artery). Specialists from the fields of sarcoma surgery, reconstructive surgery, orthopedics, traumatology, and neurosurgery were involved in this complex operation. The patient was treated with both neoadjuvant and adjuvant chemotherapy.

### 3.3. Categorization of Bone Tumor Surgery Complexity

To better categorize the complexity of individual surgeries, we established four categories, using the collected data as a reference dataset. Each of the categories contained approximately one-quarter of the registered patients (Table 3).

As previously elucidated in the methodology, the present proposition delineates a framework for categorizing patients intended for implementation in routine clinical settings. For instance, careful consideration is warranted as to whether individuals assigned to category 4 should consistently undergo treatment within specialized clinical facilities.

**Figure 5:**
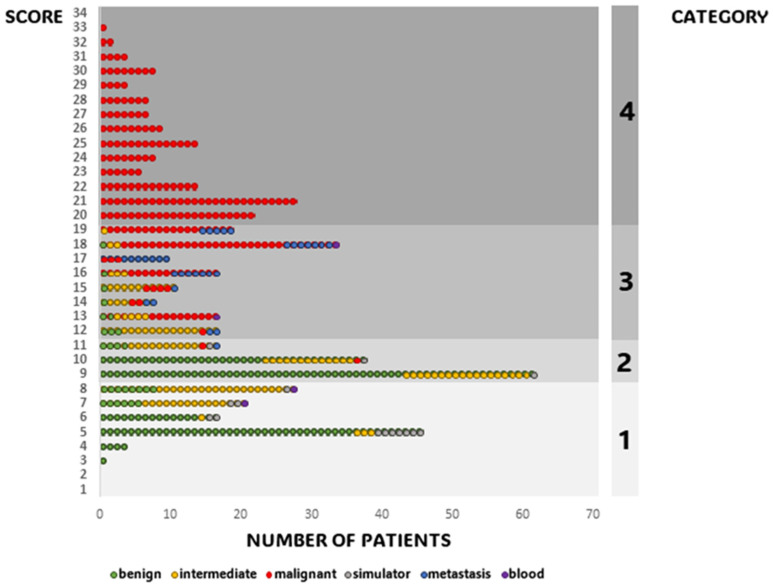
Graphical representation of individual scores and their allocation to the categories of complexity. Each point represents a patient. The malignancy of the tumors is shown in colors, as described.

Category 1 consisted of patients scoring below 9 points, representing a wide range of scores and comprising 117 patients (23.4% of the total). Within this category, patients had diverse diagnoses, including benign and intermediate tumors, as well as tumor simulators. Category 2 encompassed patients with scores ranging from 9 to 11 points, which was a relatively narrow range, but still accounted for 117 patients (23.4%). The majority of patients falling in this range had benign or intermediate diagnoses, but a few had malignant diagnoses. Patients with scores between 12 and 19 points were placed in Category 3, which included 133 patients (26.5%). Finally, Category 4, the highest category, included 134 patients (26.7%), exclusively including patients with malignant diagnoses (refer to Figure 5).

### 3.4. Validation of Complexity Scores with Case Mix Index

**Figure 6:**
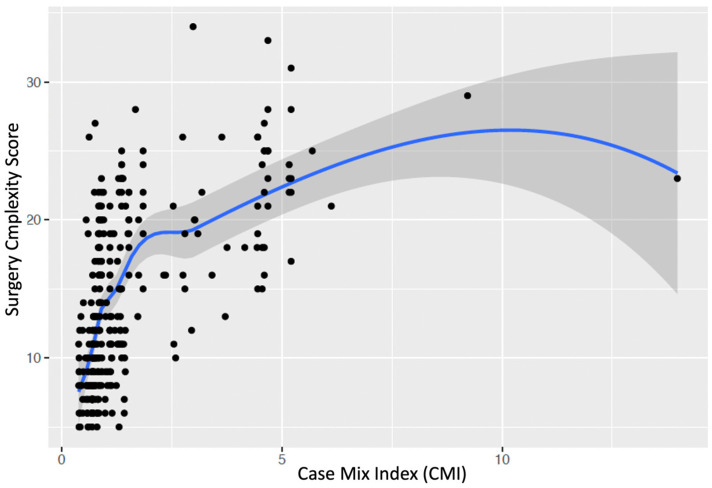
Comparison between Case Mix Index (CMI) and surgical complexity scores using Loess smoothing.

In examining the association between our newly established Surgical Complexity Score and the traditional Case Mix Index (CMI) through Loess smoothing analysis, we observed a nuanced relationship, indicative of the complexity score’s granularity (Figure 6). The analysis revealed a steep initial ascension in the complexity score corresponding to lower CMI values, which implies that our complexity score is capable of discerning surgical case variances with a high degree of sensitivity, especially at lower and medium levels of case mix severity. As CMI values increased, the complexity score demonstrated a plateau, suggesting that our score may provide a more detailed stratification of surgical cases than what is reflected by CMI alone. This plateau also points to a potential ceiling effect in the CMI’s ability to differentiate complex cases at higher severity levels. Importantly, the wide confidence intervals at the extremities of the CMI range caution against overinterpreting the complexity score’s behavior at these points, due to sparser data. Overall, these findings emphasize the added value of the Surgical Complexity Score in capturing the intricacies of case complexity, thereby offering a refined tool that surpasses the granularity of CMI, particularly in the lower-to-mid-range of case severities, where clinical decision-making and resource allocation could be most impacted.

## 4. Discussion

This study primarily aimed to develop and validate the Bone Tumor Surgery Complexity Score (BT-SCS), a comprehensive tool designed for assessing the complexity of bone tumor surgeries. Our findings indicate that the BT-SCS, encompassing patient characteristics, tumor biology, and surgical parameters, offers a nuanced and multi-dimensional approach to understanding surgical case complexity. By systematically analyzing 501 cases, we demonstrated the practicality and relevance of the BT-SCS in categorizing surgical complexity, which is crucial for optimized surgical planning and resource allocation in bone tumor treatments, as supported by current research in the field [11,26].

The practical application of the Bone Tumor Surgery Complexity Score (BT-SCS) within clinical settings is multifaceted. Its incorporation into surgical planning processes allows for a more stratified approach to case management, supporting clinical decision-making by quantifying the anticipated complexities of each surgical intervention. Consequently, it provides a framework for the judicious allocation of healthcare resources, potentially mitigating risk and optimizing patient flow. Importantly, ongoing discussions within the surgical and oncological communities suggest that the BT-SCS can refine existing benchmarks for surgical complexity, such as the CMI, by providing a more granular assessment of surgical needs. In terms of patient care, the BT-SCS can guide the referral process, ensuring that patients with complex bone tumor surgeries are directed to specialized networks equipped with the necessary expertise and resources to effectively handle such cases. Moreover, the BT-SCS stands as a benchmarking tool, enabling the comparison of surgical outcomes across different institutions and fostering an environment of continuous improvement in patient care. This tool’s potential for impacting patient outcomes emphasizes the necessity for its integration into routine clinical protocols and training programs for surgical teams.

The definition of surgical case complexity in our study comprehensively incorporates a range of factors, including preoperative considerations, intraoperative challenges, and postoperative outcomes. This holistic approach acknowledges the multifaceted nature of surgical procedures, particularly in the treatment of bone tumors [1]. By encompassing these diverse aspects, the Bone Tumor Surgery Complexity Score (BT-SCS) provides a more accurate and thorough assessment, crucial for preoperative planning, intraoperative decision-making, and postoperative care. Our work highlights the importance of a broad perspective on surgical complexity, which is essential in guiding both clinical decision-making and resource allocation, thereby enhancing patient care in orthopedic oncology, which aligns with the evolving paradigms in surgical assessment and patient care [27].

The BT-SCS scoring system was meticulously developed to provide a quantitative measure of surgical complexity in bone tumor cases. We are continually refining the system based on feedback from its practical applications, highlighting areas where additional data could enhance its precision and reliability. This methodology not only ensures clarity and consistency in scoring but also aligns with the real-world complexity encountered in clinical practice, thereby enhancing the score’s applicability and validity, as emphasized in recent studies [28,29].

The development of the BT-SCS epitomizes the shifting paradigm in surgical evaluation, moving from volume-based to complexity-based assessments. As we further validate the BT-SCS against additional clinical outcomes, it becomes clear that this tool is crucial for advancing precision medicine in orthopedic oncology, facilitating surgical decisions tailored to individual needs.

The categorization of surgical cases into four distinct groups in our study was based on the BT-SCS, which was structured to reflect varying levels of complexity in bone tumor surgeries. This grouping, independent of direct patient outcomes, was strategically chosen to facilitate clearer understanding and communication within clinical settings. It enables healthcare professionals to quickly assess and allocate resources according to the anticipated complexity of each case. These operational insights are critical as we continue to enhance the scoring system’s design and utility in clinical practice.

The Bone Tumor Surgery Complexity Score (BT-SCS) offers significant utility in surgical planning and resource allocation, facilitating a structured approach to assessing and managing bone tumor surgeries. Recognizing its limitations, such as potential biases from its retrospective design and the inherent subjectivity in any scoring system, is vital. Future research should focus on expanding the applicability of the BT-SCS in diverse settings, increasing sample diversity, and incorporating outcome metrics to enhance the score’s comprehensiveness and predictive accuracy. Continuous refinement, based on emerging research and medical community feedback is essential for maintaining the score’s relevance and utility in evolving clinical scenarios.

Future enhancements of the BT-SCS should include integrating machine learning techniques and patient outcome data to refine the categorization process. These technological advancements will enable more adaptive and responsive updates to the scoring system, accommodating changes in surgical techniques and patient demographics.

## 5. Conclusions

The development and validation of the Bone Tumor Surgery Complexity Score (BT-SCS) underscore its utility as a pivotal tool in the paradigm shift from volume-based to complexity-based assessments in bone tumor treatments. By providing a quantifiable, multi-dimensional approach to surgical case complexity, the BT-SCS enhances surgical planning, facilitates optimized resource allocation, and could potentially lead to improved patient care by enabling precise, tailored surgical interventions. Its development, through a meticulous consensus process among experienced sarcoma surgeons and a rigorous validation against the established Case Mix Index, illustrates the BT-SCS’s robustness and applicability in clinical practice. The prospective use of this tool in diverse clinical settings promises to support decision-making, improve benchmarking processes, and contribute to a value-based healthcare model. Future research endeavors should aim to further integrate patient outcomes into the BT-SCS framework, enhancing its predictive accuracy and clinical utility. As precision medicine continues to evolve, the BT-SCS is poised to become an essential component of patient-centered care, embodying the principles of modern, value-based healthcare systems.

## Figures and Tables

**Table 1:** BT-SCS system, indicating the weighting of each parameter.

				Points	Maximum
Patient’s Age	≤17 years			1	
	18–64 years			0	
	≥65 years			1	1
Histology/Grading	Benign			1	
	Simulator			1	
	Intermediate			2	
	hematologic tumors of bone (incl. myeloma and lymphoma)			3	
	Metastasis			5	
	Malignant	G1		5	
	Malignant	G2		6	
	Malignant	G3		7	7
Prior History *	Preoperative radiotherapy			2	
	Preoperative chemotherapy			2	
	Prior unplanned Excision (UE)			2	6
Anatomical Location	Extremities	Upper extremity		1	
	Extremities	Lower extremity		1	
	Trunk			1	
	Spine/pelvis			3	3
Size of Lesion	Appendicular skeleton, trunk, skull, and facial bone lesions				
	No evidence of primary tumor			0	
	≤8 cm			1	
	>8 cm			2	
	Discontinuous tumors in primary bone site			3	
	Pelvis				
	Tumor confined to one pelvic segment with no extraosseous extension				
		Tumor ≤ 8 cm in greatest dimension		1	
		Tumor > 8 cm in greatest dimension		2	
	Tumor confined to one pelvic segment with extraosseous extension or two segments without extraosseous extension				
		Tumor ≤ 8 cm in greatest dimension		2	
		Tumor > 8 cm in greatest dimension		3	
	Tumor spanning two pelvic segments with extraosseous extension				
		Tumor ≤ 8 cm in greatest dimension		3	
		Tumor > 8 cm in greatest dimension		4	
	Tumor spanning three pelvic segments or crossing the sacroiliac joint				
		Involves sacroiliac joint and extends medial to the sacral neuroforamen		4	
		Encasement of external iliac vessels or presence of gross tumor thrombus in major pelvic vessels		5	
	Spine				
	One vertebral segment or two adjacent vertebral segments			1	
	Confined to three adjacent vertebral segments			2	
	Four or more adjacent segments or any nonadjacent vertebral segments			3	
	Extension into spinal canal			3	
	Evidence of gross vascular invasion or tumor thrombus in the great vessels			3	5
Type of Bone Resection	Biopsy, radiofrequency ablation, HIFU, cryotherapy, curettage/intralesional/piece-meal/decompression surgery			1	
	Resection of muscles			1	
	Resection of vessels			2	
	Resection of nerves			1	
	Resection of tendons			1	
	Hemicortex resection			2	
	Transarticular bone resection			4	
	Joint-sparing (intercalary segmental) resection			2	
	Extra-articular resection			5	
	En bloc vertebrectomy			5	
	Rotationplasty			6	
	Epiphysiolysis			2	
	Resection–replantation			6	
	Tikhoff–Linberg resection			6	
	Internal hemipelvectomy	Type I		6	
	Internal hemipelvectomy	Type II		8	
	Internal hemipelvectomy	Type III		6	
	Internal hemipelvectomy	Type IV		7	
	Amputations	Forequarter		6	
	Amputations	External hemipelvectomy	Transacetabular	6	
	Amputations	External hemipelvectomy	Transiliac	6	
	Amputations	External hemipelvectomy	Transhemisacral without spinopelvic dissociation	6	
	Amputations	External hemipelvectomy	Transhemisacral with spinopelvic dissociation	8	
	Amputations	External hemipelvectomy	Hemicorporectomy	10	
	Amputations	Upper extremity	Transarticular shoulder/elbow—humerus	4	
	Amputations	Upper extremity	Forearm/wrist	3	
	Amputations	Upper extremity	Hand/finger	2	
	Amputations	Lower extremity	Hip disarticulation	5	
	Amputations	Lower extremity	Thigh/knee disarticulation	5	
	Amputations	Lower extremity	Leg	3	
	Amputations	Lower extremity	Foot/digits	2	26 **
Type of Reconstruction	Cementation/ORIF			2	
	Artificial bone substitute/autograft/allograft chips			2	
	Bulk allograft			3	
	Pasteurized autograft			3	
	Nonvascularized fibula			4	
	Vascularized fibula based on fibular artery			6	
	Vascularized fibula based on tibialis anterior			6	
	Pedicled-tissue transfer			3	
	Free-tissue transfer			4	
	Skin/mesh graft			1	
	Nerve, vessel, and lymphovenous reconstruction			4	
	Tendon reconstruction			2	
	Conventional prosthesis			2	
	Modular tumor prosthesis			4	
	Custom-made prosthesis			6	
	Growing prosthesis			6	
	Spinal instrumentation with pedicle screws/rods			3	
	Arthrodesis			3	
	Pseudarthrosis/flail joint/cement spacer			2	
	Cañadell distraction epiphysiolysis			4	
	Distraction osteogenesis			5	
	Goretex, Trevira, etc.			2	
	Stump after amputation			2	18 ***
Number of Involved Disciplines ****	One discipline			0	
	Two disciplines			1	
	Three disciplines			2	
	Four disciplines			3	
	Five or more disciplines			4	4
Total				max.	70

* The points in the section “prior history” can be added together, resulting in a maximum score of 6 in this field. ** Max. 3 different types of resections can be added together. *** Max. 3 different types of reconstructions can be added together. **** If one single surgeon is a sarcoma surgeon, but also has the credentials for vascular reconstruction, then 2 disciplines are registered.

**Table 2:** This table characterizes the independent patient cohort for validation purposes of the BS- and STS-SCS.

Parameter	Number of Patients
Patients (m/f)	363 (205/158)
Age/range	51 (7–88)
Soft tissue tumors	284
Bone tumors	79
Benign	117
Intermediate	83
Malignant	163
SST Cat 1 ^1^	18
SST Cat 2	55
SST Cat 3	88
SST Cat 4	106
BS Cat 1 ^2^	7
BS Cat 2	23
BS Cat 3	26
BS Cat 4	14
CMI ^3^	
- mean (SD)	1.5 (SD)
- range	0.38, 13.98
- median (IQR)	0.86 (0.71, 1.38)

^1^ SST = soft tissue tumors; ^2^ BS = bone tumors; ^3^ CMI = Case Mix Index.

**Table 3:** Division of surgeries into four categories.

Category	Complexity Score	Number of Patients	Percentage (%)
1	≤8	117	23.4%
2	9–11	117	23.4%
3	12–19	133	26.5%
4	≥20	134	26.7%

## Data Availability

The data presented in this study are available on request from the corresponding author due to privacy.
